# Impaired β-glucocerebrosidase activity and processing in frontotemporal dementia due to progranulin mutations

**DOI:** 10.1186/s40478-019-0872-6

**Published:** 2019-12-23

**Authors:** Andrew E. Arrant, Jonathan R. Roth, Nicholas R. Boyle, Shreya N. Kashyap, Madelyn Q. Hoffmann, Charles F. Murchison, Eliana Marisa Ramos, Alissa L. Nana, Salvatore Spina, Lea T. Grinberg, Bruce L. Miller, William W. Seeley, Erik D. Roberson

**Affiliations:** 1 Departments of Neurology and Neurobiology, Center for Neurodegeneration and Experimental Therapeutics, Alzheimer’s Disease Center, Evelyn F. McKnight Brain Institute, University of Alabama at Birmingham, Birmingham, AL USA; 2 1825 University Blvd., SHEL 1106, Birmingham, AL 35294 USA; 3 Department of Biostatistics, University of Alabama at Birmingham, Birmingham, AL USA; 4 Department of Psychiatry, David Geffen School of Medicine, University of California Los Angeles, Los Angeles, CA USA; 5 Department of Neurology, Memory & Aging Center, UCSF Weill Institute for Neurosciences, University of California, San Francisco, San Francisco, CA USA; 6 Department of Pathology, University of California, San Francisco, San Francisco, CA USA; 7 1825 University Blvd., SHEL 1110, Birmingham, AL 35294 USA

**Keywords:** Progranulin, Lysosome, β-Glucocerebrosidase, Glycosphingolipid, Frontotemporal dementia, Neuronal Ceroid Lipofuscinosis

## Abstract

Loss-of-function mutations in progranulin (*GRN*) are a major autosomal dominant cause of frontotemporal dementia. Most pathogenic *GRN* mutations result in progranulin haploinsufficiency, which is thought to cause frontotemporal dementia in *GRN* mutation carriers. Progranulin haploinsufficiency may drive frontotemporal dementia pathogenesis by disrupting lysosomal function, as patients with *GRN* mutations on both alleles develop the lysosomal storage disorder neuronal ceroid lipofuscinosis, and frontotemporal dementia patients with *GRN* mutations (FTD-*GRN*) also accumulate lipofuscin. The specific lysosomal deficits caused by progranulin insufficiency remain unclear, but emerging data indicate that progranulin insufficiency may impair lysosomal sphingolipid-metabolizing enzymes. We investigated the effects of progranulin insufficiency on sphingolipid-metabolizing enzymes in the inferior frontal gyrus of FTD-*GRN* patients using fluorogenic activity assays, biochemical profiling of enzyme levels and posttranslational modifications, and quantitative neuropathology. Of the enzymes studied, only β-glucocerebrosidase exhibited impairment in FTD-*GRN* patients. Brains from FTD-*GRN* patients had lower activity than controls, which was associated with lower levels of mature β-glucocerebrosidase protein and accumulation of insoluble, incompletely glycosylated β-glucocerebrosidase. Immunostaining revealed loss of neuronal β-glucocerebrosidase in FTD-*GRN* patients. To investigate the effects of progranulin insufficiency on β-glucocerebrosidase outside of the context of neurodegeneration, we investigated β-glucocerebrosidase activity in progranulin-insufficient mice. Brains from *Grn*^*−/−*^ mice had lower β-glucocerebrosidase activity than wild-type littermates, which was corrected by AAV-progranulin gene therapy. These data show that progranulin insufficiency impairs β-glucocerebrosidase activity in the brain. This effect is strongest in neurons and may be caused by impaired β-glucocerebrosidase processing.

## Introduction

Loss-of-function mutations in progranulin (*GRN*) are an autosomal dominant cause of frontotemporal dementia (FTD), causing as much as 5–10% of FTD cases [5, 16, 20]. Most of these mutations cause progranulin haploinsufficiency, with *GRN* carriers having less than half of normal circulating progranulin levels [19]. In rare cases, individuals have been found with loss-of-function *GRN* mutations on both alleles, resulting in an almost complete loss of progranulin [2, 59]. Instead of FTD, these individuals develop a lysosomal storage disorder, neuronal ceroid lipofuscinosis (NCL), characterized by neurodegeneration and accumulation of lysosomal storage material [2, 59]. Brains from FTD-*GRN* patients accumulate similar storage material as NCL patients [25, 67], so lysosomal dysfunction may be a key mechanism of FTD-*GRN* pathogenesis.

Understanding how the lysosome is impaired by progranulin insufficiency may enable targeted therapies for FTD due to *GRN* mutations. Emerging data indicate that progranulin may regulate activity of lysosomal enzymes involved in sphingolipid metabolism. Progranulin interacts with and facilitates lysosomal localization of prosaposin [45, 73, 74], a pro-protein which is cleaved into saposin fragments that serve as critical co-factors for sphingolipid-metabolizing enzymes [57, 58]. Progranulin insufficiency disrupts prosaposin trafficking in the brain [74]. In some cell types, progranulin regulates trafficking and activity of at least two enzymes involved in sphingolipid metabolism, β-hexosaminidase A (HexA) [14] and β-glucocerebrosidase (GCase) [29, 30]. We therefore hypothesized that progranulin insufficiency would impair activity of sphingolipid-metabolizing enzymes in the brain. To test this hypothesis, we measured enzyme activity, levels, and post-translational modifications in inferior frontal gyrus of FTD-*GRN* patients and frontal cortex of progranulin-insufficient mice. We assessed the interaction of progranulin with GCase in cultured cells and investigated the effects of AAV-progranulin gene therapy on GCase enzyme activity in *Grn*^*−/−*^ mice.

## Materials and methods

### Patient brain samples

Post-mortem brain samples were provided by the Neurodegenerative Disease Brain Bank at the University of California, San Francisco. Brains were donated with the consent of the patients or their surrogates in accordance with the Declaration of Helsinki, and the research was approved by the University of California, San Francisco Committee on Human Research. Tissue blocks were dissected from the inferior frontal gyrus of 5 controls and 7 patients with FTD-*GRN*. All patients with FTD-*GRN* carried a pathogenic variant in *GRN* and had FTLD-TDP, Type A identified at autopsy, except one (case 7) discussed more extensively in the text. More extensive patient characteristics are provided in Table 1. Clinical and neuropathological diagnoses were made using standard diagnostic criteria [23, 35, 36, 43, 53]. *GBA* sequencing data were available for a subset of patients. These samples were screened by targeted sequencing of a panel of genes implicated in neurodegenerative disorders [52]. Coding and exon-intron boundary regions of the *GBA* gene were screened for pathogenic variants classified according to the American College of Medical Genetics and Genomics and the Association for Molecular Pathology guidelines [54]. All patients with available data (1 control and 4 FTD-*GRN* patients) were negative for pathogenic *GBA* variants.

**Table 1:** Cases studied

Case	Group	Sex	Age at death	PMI (hours)	Clinical Diagnosis	Primary Path Diagnosis^a^	Alzheimer’s disease neuropathologic change	Thal Amyloid Plaque Phase	Braak Neurofibrillary Degeneration Stage	CERAD Neuritic Plaque Score	LBD Stage	*GBA* Variants	Other Path Diagnosis^b^
1	Ctrl	F	86	6.4	Control	N/A	Not	0	2	Absent	Brainstem predominant	nd	AGD, limbic; VBI
2	Ctrl	F	81	30.3	MCI, amnestic	PART	Low	1	2	Absent	0	negative	None
3	Ctrl	M	76	8.2	Control	N/A	Low	1	2	Sparse	0	nd	AGD, limbic
4	Ctrl	M	77	4.9	MCI, executive	AGD	Low	2–3	2	Sparse	0	nd	VBI, microinfarct in cerebellar folia
5	Ctrl	F	86	7.8	Control	N/A	Low	1	2	Moderate	0	nd	VBI, microinfarcts in claustrum and angular gyrus; AGD
6	*GRN*	F	59	9.5	CBS	FTLD-TDP-A	Low	2	1	Frequent	0	negative	CAA
7	*GRN*	M	66	10.1	DLB,? bvFTD	LBD	Not	0	2	Absent	Diffuse neocortical type	negative	Incipient FTLD-TDP-A
8	*GRN*	M	64	7.2	bvFTD	FTLD-TDP-A	Not	0	2	Absent	0	nd	None
9	*GRN*	M	72	23.8	PPA-mixed	FTLD-TDP-A	Not	0	0	Absent	0	negative	Subdural hematoma
10	*GRN*	M	74	30.9	nfvPPA, CBS	FTLD-TDP-A	Intermediate-High	2–5	4–5	Moderate	0	negative	None
11	*GRN*	F	73	20.7	nfvPPA, CBS	FTLD-TDP-A	Not	0	2	Absent	0	nd	None
12	*GRN*	F	66	7.4	bvFTD	FTLD-TDP-A	Low	1	0	Sparse	0	nd	VBI, microinfarcts in putamen

*PMI*, postmortem interval, *MCI* Mild cognitive impairment, *CBS* Corticobasal syndrome, *DLB* Dementia with Lewy bodies, *bvFTD* Behavioral variant frontotemporal dementia, *PPA* Primary progressive aphasia, *nfv* nonfluent variant, *PART* Primary age-related tauopathy, *AGD* Argyrophilic grain disease, *LBD* Lewy body disease, *VBI* Vascular brain injury, *CAA* Cerebral amyloid angiopathy, *nd* = data not available

^a^Disease considered most likely to explain the clinical syndrome

^b^No subject had limbic TDP-43 proteinopathy (except those with FTLD-TDP)

### Animals

*Grn*^*−/−*^ mice were generated and crossed onto a C57BL/6 J background as previously described [18, 37]. Wild-type, *Grn*^*+/−*^*,* and *Grn*^*−/−*^ mice for this study were generated by crossing male and female *Grn*^*+/−*^ mice. The resulting littermates were used for all mouse studies, and both male and female littermates were used. The mice were housed in a barrier facility accredited by the Association for Assessment and Accreditation of Laboratory Animal Care, under conditions previously described [4]. All experiments were approved by the Institutional Animal Care and Use Committee of the University of Alabama at Birmingham.

### Fibroblasts

Fibroblast lines were purchased from the NIGMS Human Genetic Cell Repository at the Coriell Institute for Medical Research. We analyzed cells from an apparently healthy line (#GM00730), a heterozygous *GBA* L444P line (#GM00878), and two homozygous *GBA* L444P lines (#GM08760 and GM00877). Fibroblasts were cultured in MEM (Corning Life Sciences) with 15% fetal bovine serum (Atlanta Biologicals) and 1% penicillin/streptomycin (ThermoFisher) at 37 °C and 5% CO_2_. Deglycosylation reactions were carried out on 25 μg of protein as described below.

### Tissue preparation

For initial western blot and lysosomal activity assays, tissue from mouse frontal cortex or human inferior frontal gyrus was homogenized in lysis buffer (50 mM Tris, 150 mM NaCl, 5 mM EDTA, 1% Triton X-100, 0.1% sodium deoxycholate) and spun at 5000 x *g* for 10 min. Protein content of the supernatant was measured by Bradford assay, and equal amounts of total protein from each sample were used for subsequent assays.

### Western blot

Samples were run on 4–12% bis-tris polyacrylamide gels (ThermoFisher), transferred to Immobilon-FL PVDF (MilliporeSigma), and probed overnight with primary antibody. The following primary antibodies were used: rabbit polyclonal GCase (#G4171, MilliporeSigma), mouse monoclonal GCase (#sc-166,407, Santa Cruz Biotechnology), cathepsin D (goat polyclonal, #sc-6486, Santa Cruz Biotechnology), LAMP-1 (mouse monoclonal, #sc-20,011, Santa Cruz Biotechnology), LAMP-2 (mouse monoclonal, #sc-18,822, Santa Cruz Biotechnology), GAPDH (mouse monoclonal, #MAB374, MilliporeSigma), Flag (mouse monoclonal, #F3165, MilliporeSigma), and progranulin (rabbit polyclonal, #40–3400, ThermoFisher). Blots were then probed with species-matched IRdye-conjugated secondary antibodies (Li-COR Biosciences) and scanned on an Odyssey scanner (Li-COR Biosciences).

### Lysosomal activity assays

Activity of β-galactosidase (β-Gal), β-hexosaminidase (total) (Hex), β-hexosaminidase A (HexA), α-galactosidase A (GLA), and β-glucocerebrosidase (GCase) was determined in tissue lysates by incubation with fluorogenic substrates at 37 °C. All reactions resulted in production of the fluorophore 4-methylumbelliferone (4-MU), and were read on a Synergy 2 plate reader (Biotek Instruments) with a 360 nm excitation wavelength and a 440 nm emission wavelength. Data were quantitated relative to a standard curve of 4-MU run on each plate and calculated as nmol 4-MU generated per hour per mg of protein loaded. For all reactions, a uniform amount of protein was loaded per well, ranging from 5 to 20 μg depending on the assay.

Activity of Hex and HexA was determined by incubation with 2 mM substrate (for β-Hex - 4-methylumbelliferyl-2-acetamido-2-deoxy-β-D-glucopyranoside, MilliporeSigma, for HexA - 4-methylumbelliferyl-2-acetamido-2-deoxy-6-sulfate-β-D-glucopyranoside, Research Products International) in 10 mM sodium citrate buffer, pH 4.2 [69]. Reactions were stopped with 0.2 M glycine, 0.2 M sodium carbonate.

Activity assays for GCase (substrate: 1 mM 4-Methylumbelliferyl β-D-glucopyranoside, MilliporeSigma), GLA (substrate: 4.3 mM 4-Methylumbelliferyl α-D-galactopyranoside, MilliporeSigma) and β-Gal (substrate: 1 mM 4-Methylumbelliferyl β-D-galactopyranoside, MilliporeSigma) were conducted in pH 4.6 citrate phosphate buffer and stopped with 0.4 M glycine, pH 10.8. For GCase, the reaction buffer included 1% bovine serum albumin, 0.25% triton X-100, 0.25% taurocholic acid, and 1 mM EDTA [44]. Specific GCase activity was confirmed by correcting for fluorescence generated in the presence of a GCase inhibitor, 0.18 mM conduritol β-epoxide (Enzo Life Sciences). Specific GLA activity was determined by inhibition of non-specific substrate cleavage by α-galactosidase B using 90 mM N-acetyl-D-galactosamine (MilliporeSigma) [44].

### Endoglycosidase H and PNGase F treatment

Tissue lysates prepared as described above were incubated with endoglycosidase H (New England Biolabs) or PNGase F (New England Biolabs) according to the manufacturer’s instructions as previously described [3]. For each sample, a control tube was run for each enzyme that contained all reaction components except for the enzyme. Reactions containing 50 μg of protein were incubated overnight at 37 °C, then processed for western blot as described above.

### Sarkosyl soluble/insoluble fractionation

Sarkosyl soluble and insoluble fractions were prepared using a previously described protocol [17]. Protein content of the fractions was determined by BCA assay (ThermoFisher), and equivalent amounts of protein from each sample were processed for western blot as described above.

### Immunostaining

Formalin-fixed blocks of inferior frontal gyrus were provided by the Neurodegenerative Disease Brain Bank at the University of California, San Francisco. Tissue blocks were cryoprotected in 30% sucrose and cut into 30 μm sections on a sliding microtome. Prior to immunostaining, sections underwent antigen retrieval in 10 mM sodium citrate, pH 6.0 at 80 °C for 2 h. Sections were then washed in PBS and immunostained as previously described using rabbit polyclonal (#G4171, MilliporeSigma) or mouse monoclonal anti-GCase antibodies (#MAB7410, R&D Systems) [50]. Sections were blocked in 10% normal goat serum, 1% milk, and 0.2% gelatin prior to overnight incubation with primary antibody diluted in 3% normal goat serum and 0.2% gelatin. After washing, sections were probed with species-matched secondary antibodies diluted in 3% normal goat serum and 0.2% gelatin. Sections were then washed and incubated with avidin-biotin complex (VectaStain Elite, Vector Laboratories). Immunostaining was detected by incubating with 3,3′-diaminobenzidene (MP Biomedicals) in 100 mM Tris, pH 7.4. All antibody incubations and washes were conducted with PBS containing 0.5% Triton X-100. Initial quantification of GCase immunolabeling was performed on sections with no counterstain, but in follow-up studies sections were counterstained with either Cresyl violet (MilliporeSigma) or hematoxylin (Fisher Scientific). For fluorescent immunostaining, sections were co-stained with a rabbit monoclonal anti-NeuN antibody (#ab177487, Abcam). GCase immunolabeling was detected with an AlexaFluor-488 anti-mouse antibody (ThermoFisher Scientific), and NeuN immunolabeling was detected with an AlexaFluor-594 anti-rabbit antibody (ThermoFisher Scientific). Fluorescently-labeled sections were stained with 1% Sudan Black (Acros Organics) to quench autofluorescence.

### Imaging and analysis

Imaging and analysis of GCase-immunostained tissue was performed by investigators blinded to experimental group. Chromogenic GCase-immunostained tissue sections were imaged at 20X on a brightfield microscope (Nikon) and analyzed using ImageJ. For each patient, 2–3 20X fields of view were captured in cortical layer III. Total GCase immunoreactivity was assessed on sections with no counterstain by measuring the area falling within a uniform intensity threshold. In a follow-up study, sections were counterstained with hematoxylin. Hematoxylin (blue) and GCase (brown) labeling were deconvoluted, and neurons were defined by using ImageJ’s Analyze Particles feature to draw ROIs. These ROIs were then overlaid onto the GCase image to define neuronal GCase immunoreactivity.

Fluorescently-stained sections were imaged at 40X on a Nikon A1R confocal. For each patient 2–4 z-stacks of 25 μm thickness were obtained using 0.5 μm z-steps. Maximum intensity projections were made of both NeuN and GCase immunoreactivity. Neuronal ROIs were drawn using ImageJ’s analyze particles feature on the NeuN images. These ROIs were overlaid onto the GCase images to measure the fluorescent intensity of each neuron.

### Cell culture and co-immunoprecipitation

HEK-293 cells (#CRL-1573, ATCC) were cultured in DMEM (Corning Life Sciences) with 10% fetal bovine serum (Atlanta Biologicals), and 1% penicillin/streptomycin (ThermoFisher) at 37 °C and 5% CO_2_. Cells were transiently transfected with a plasmid expressing human *GBA1* with a C-terminal myc-flag tag (#RC216061, Origene), and/or a construct expressing human progranulin with an N-terminal HA tag inserted after the signal peptide in the previously described CIGW vector [4, 60]. After 48 h, the cells were harvested in lysis buffer (50 mM Tris, 150 mM NaCl, 5 mM EDTA, 1% Triton X-100, 0.1% sodium deoxycholate) and centrifuged 10 min at 5000 x *g*. HA-tagged progranulin was immunoprecipitated from 100 μg of lysate protein using 2.5 μg of anti-HA tag antibody (mouse monoclonal, #sc-7392, Santa Cruz Biotechnology) as described above. Immunoprecipitates were blotted for Flag tag (mouse monoclonal, #F3165, MilliporeSigma) to detect co-immunoprecipitated GCase, and for progranulin to confirm successful pull down of HA-tagged progranulin (rabbit polyclonal, #40–3400, ThermoFisher).

### Proximity ligation assays

HEK-293 cells were cultured on 12 mm No. 1 glass coverslips (Carolina Biological) coated with 0.1 mg/mL Poly-D-Lysine (MilliporeSigma) and 0.02 mg/mL laminin (MilliporeSigma), then transfected with *GBA*-myc-Flag and HA-*GRN* plasmids as described above. Forty-eight hours after transfection, cells were fixed in 4% paraformaldehyde and 4% sucrose. The coverslips were incubated overnight at 4 °C with anti-HA (rabbit monoclonal, #3274, Cell Signaling Technologies) and anti-Flag (goat polyclonal, #ab1257, Abcam) antibodies, and probed the next day with a Duolink proximity ligation kit (MilliporeSigma) with donkey anti-rabbit and donkey anti-goat probes using the manufacturer’s protocol. All coverslips were imaged with an epifluorescent microscope (Nikon) at 60x.

### Lipidomics

Levels of glucosylceramide isoforms and glucosylsphingosine were measured at the Lipidomics Shared Resource at the Medical University of South Carolina. Shavings from frozen tissue blocks were homogenized (lysate buffer: 0.25 M sucrose, 25 mM KCl, 0.5 mM EDTA, 50 mM Tris, pH 7.4), and protein content was determined by Bradford assay. Lipids were extracted from lysate containing 1 mg of total protein, and were measured with high-performance liquid chromatography/mass spectrometry as previously described [8].

### AAV administration

The effects of AAV-progranulin on GCase activity in *Grn*^*−/−*^ mice were measured in ventral striatum samples from mice used in a previous study of AAV-progranulin [4]. A second group of mice was injected with AAV-progranulin or AAV-GFP by infusion in the medial prefrontal cortex as previously described [4]. These mice were treated at 3–6 months of age and euthanized for tissue collection 4 weeks later.

### Experimental design & statistics

Lysosomal enzyme activity, protein levels, and chromogenic immunostaining in patient samples were analyzed by *t*-test. The intensity of fluorescent GCase immunolabeling was analyzed by mixed-effects regression models with random effects intercept term blocking on subjects to account for the repeated measures. Linear mixed-models were used to compare mean intensities between groups while quantile regression was used to compare deciles. Levels of glucosylceramide isoforms were analyzed by MANOVA. Lysosomal enzyme activity in mice was analyzed by one-way ANOVA. Main effects of genotype were followed by Dunnett’s post-hoc test to compare *Grn*^*+/−*^ and *Grn*^*−/−*^ mice to wild-type. The effects of AAV-progranulin in ventral striatum samples were analyzed by two-way ANOVA with factors of genotype and virus. The effects of AAV-progranulin across multiple brain regions were analyzed by repeated-measures ANOVA with factors of genotype, virus, and brain region, followed by individual repeated measures ANOVA for each genotype with factors of virus and brain region. These two-way repeated measures ANOVA were followed by Sidak’s post-hoc test to analyze the effect of virus within each brain region. MANOVA and three-way repeated measures ANOVA were performed with SPSS 24 (IBM), Mixed-model regression was performed with R with additional utility from the lme4 and lqmm packages, and all other analyses were performed with Graphpad Prism 7 (GraphPad). For all analyses, two-tailed *p* values were calculated, with α set at 0.05.

## Results

### Increased HexA and decreased GCase activity patients with FTD-*GRN*

**Fig. 1:**
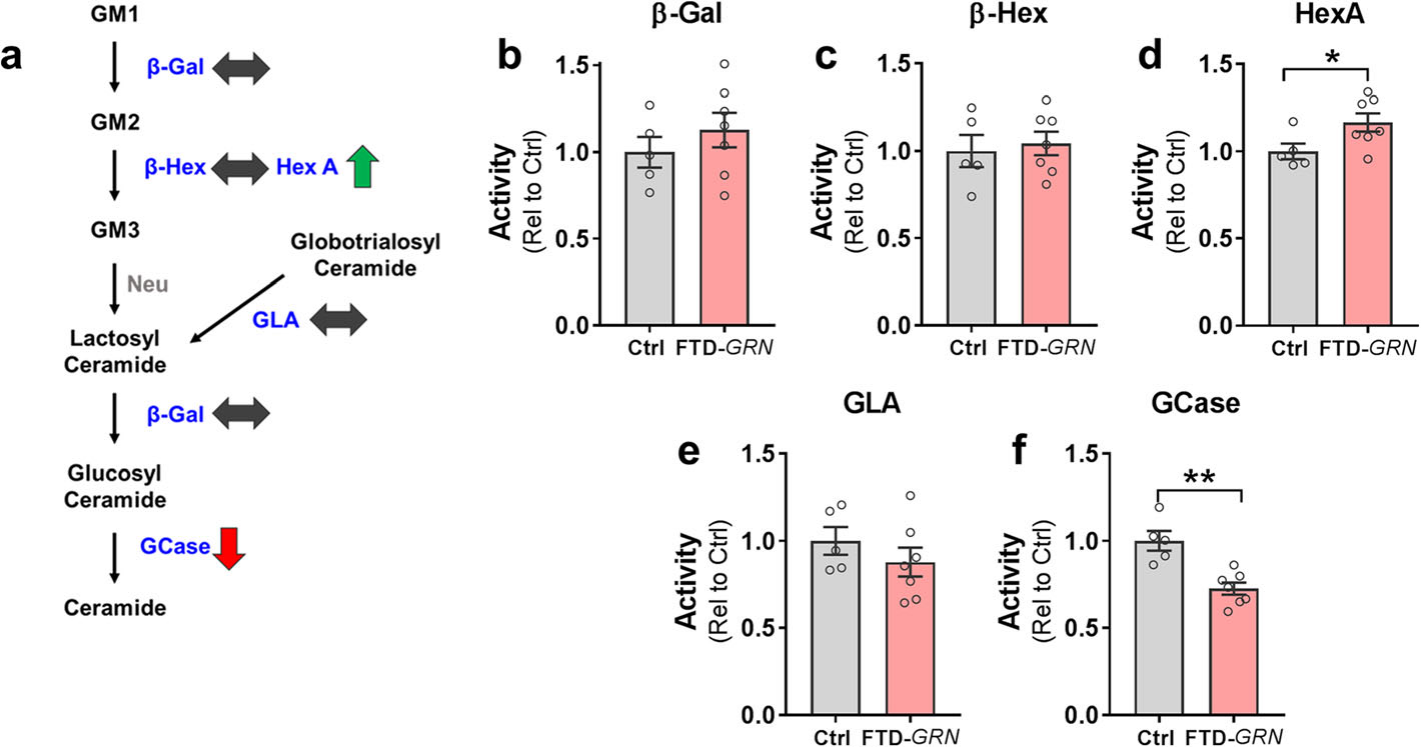
Deficits in GCase Activity in Inferior Frontal Gyrus from Patients with FTD-*GRN*. Lysates from inferior frontal gyrus of controls and FTD-*GRN* patients were analyzed for activity of sphingolipid-metabolizing enzymes. **a,** Simplified diagram of metabolism of gangliosides, with a summary of observed phenotypes in FTD-*GRN* cases. Lipids are shown in black, with the enzymes that metabolize each lipid shown in blue. GM1, 2, and 3 = GM1, 2, and 3 ganglioside, β-Gal = β-galactosidase, β-Hex = β-Hexosaminidase, HexA = β-Hexosaminidase A, GLA = α-galactosidase A, GCase = β-glucocerebrosidase, Neu = neuraminidase (activity not measured in this study). **b–f**, Enzymatic activity in FTD-*GRN* patient brains as measured by fluorogenic assays for **b**, β-Gal, **c**, β-Hex, **d**, HexA, which was increased (* = *t* test, *p* = 0.0474), **e**, GLA, and **f**, GCase, which was decreased (** = *t* test, *p* = 0.0015). *n* = 5 controls, 7 FTD-*GRN*

We hypothesized that progranulin insufficiency would impair the activity of sphingolipid-metabolizing enzymes in the brain. To test this hypothesis, we measured activity of β-galactosidase (β-Gal), total β-hexosaminidase (β-Hex), β-hexosaminidase A (HexA), α-galactosidase A (GLA), and β-glucocerebrosidase (GCase) in postmortem samples of inferior frontal gyrus from controls and patients with FTD-*GRN* (Fig. 1a). We detected an increase in HexA activity (Fig. 1d) and a reduction in GCase activity (Fig. 1f), but no change in β-Gal, GLA, or total β-Hex activity in FTD-*GRN* patients relative to controls.

This pattern of changes in enzyme activity was not consistent with a generalized impairment of saposin-dependent enzymes [58], as β-Gal and GLA require saposin B for optimal activity but were unchanged in FTD-*GRN* patients. Instead, the selective changes in HexA and GCase activity in FTD-GRN patients may be related to their reported interaction with progranulin [14, 29, 30]. The opposite directionality of the changes in HexA and GCase was somewhat surprising. Progranulin may facilitate the processing and lysosomal trafficking of both enzymes, which could result in reduced activity under progranulin-insufficient conditions [14, 29, 30]. However, we and others have observed increased HexA activity in brains from progranulin-insufficient mice [3, 33, 61, 62], perhaps indicating cell-type–specific effects of progranulin-insufficiency on enzyme function. To gain further insight into the mechanisms underlying the altered HexA and GCase activity, we investigated how the changes in enzyme activity related to enzyme levels in brains of FTD-*GRN* patients.

### Increased HexA protein levels in patients with FTD-*GRN*

We observed elevated HexA protein levels in FTD-*GRN* patients relative to controls (Additional file 1: Figure S1a). This increase in HexA levels is consistent with a general increase in lysosomal proteins in FTD-*GRN* patients [25] and progranulin-insufficient mice [24, 25, 33, 61, 62]. Consistent with these prior reports, we also observed elevated LAMP-1 and LAMP-2 levels, and a trend for increased mature cathepsin D levels in FTD-*GRN* patients (Additional file 1: Figure S1). Given the general increase in lysosomal proteins in FTD-*GRN* patients, the increase in HexA and other lysosomal proteins may be a compensatory response to lysosomal dysfunction caused by progranulin haploinsufficiency.

### Abnormal GCase processing in FTD-*GRN*

**Fig. 2:**
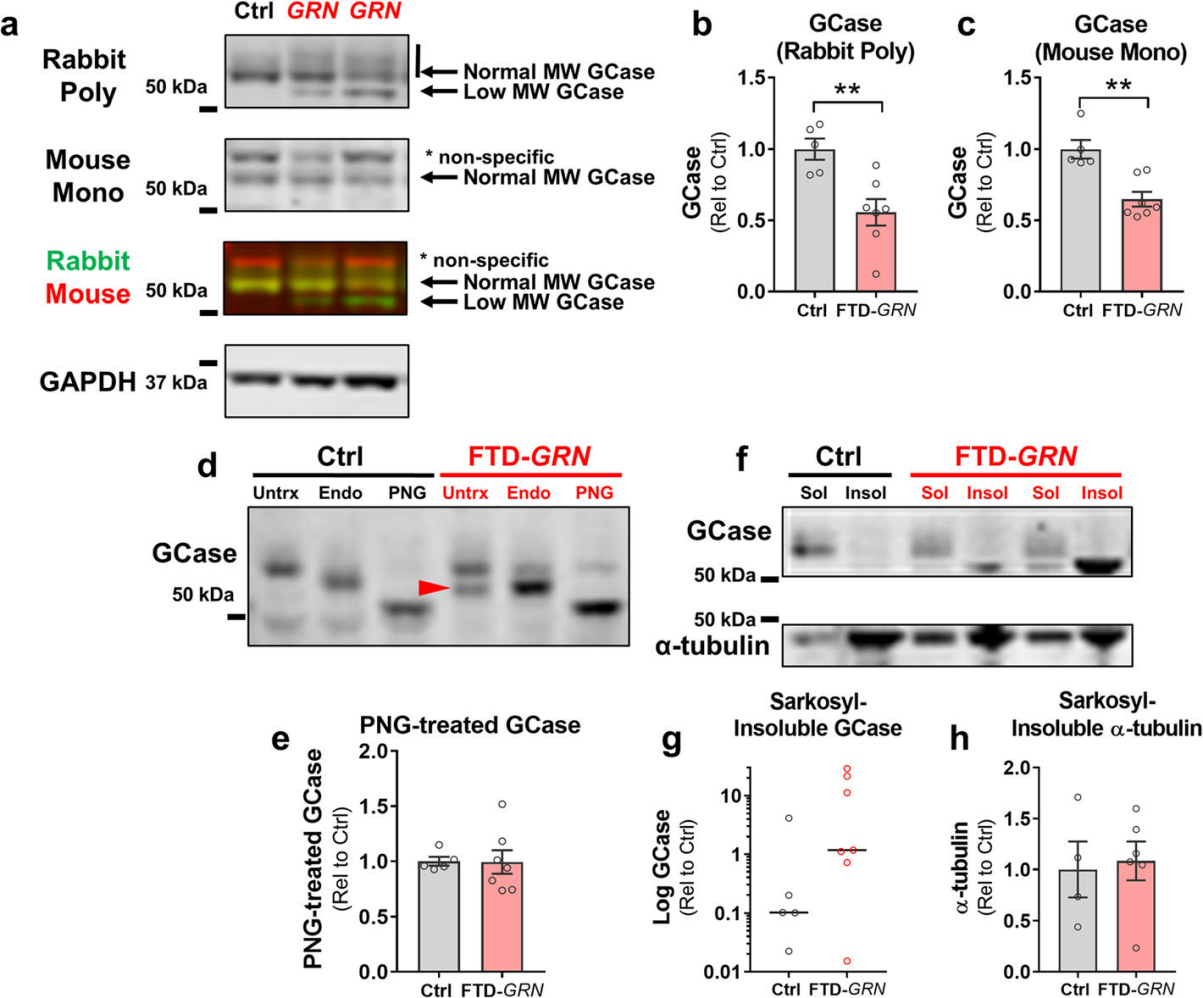
Lower Levels of Mature GCase Protein and Accumulation of Incompletely Glycosylated GCase in Brains from FTD-*GRN* Patients. **a–c**, Lysates of inferior frontal gyrus from FTD-*GRN* cases had lower levels of normal molecular-weight GCase protein than controls as detected by a rabbit polyclonal anti-GCase antibody (**b**, *t* test, *p* = 0.0059), and a mouse monoclonal anti-GCase antibody (**c**, *t* test, *p* = 0.0017). The polyclonal antibody also detected a lower molecular weight band that was present in all but one FTD-*GRN* case, but rarely in controls. **d**, To determine whether this lower molecular weight band could represent incompletely glycosylated GCase, lysates were treated with Endo H or PNGase F. The lower-molecular weight GCase band (red arrowhead) was approximately the same molecular weight as Endo H-treated GCase. Complete removal of glycosylation with PNGase F collapsed the multiple GCase bands into one band just over 50 kDa, indicating that the low-molecular weight form of GCase detected by the polyclonal antibody may consist of incompletely glycosylated GCase. The higher molecular weight band detected by the mouse monoclonal antibody was determined to be non-specific because it did not shift with PNGase F treatment (data not shown). **e**, Unlike normal molecular weight GCase, there were no group differences in deglycosylated (PNGase F-treated) GCase (*t* test, *p* = 0.9594), showing that while FTD-*GRN* brains contain less normal molecular weight, active GCase than controls, they have similar total levels of GCase protein. **f**, The low-molecular weight form of GCase accumulated in the sarkosyl-insoluble fraction of FTD-*GRN* brains, while normal molecular weight GCase was only detectable in the soluble fractions. Levels of sarkosyl-insoluble GCase did not statistically differ between controls and FTD-*GRN* patients (**g**, Mann-Whitney test, *p* = 0.1490), but the insoluble band was detectable in 6/7 FTD-*GRN* patients, but only reliably detectable in 1/5 controls. This stands in contrast to α-tubulin (**f**, **h**), which did not differ between groups. Levels of insoluble GCase were very high in some FTD-*GRN* patients, so data in **g** are shown in log scale. *n* = 5 controls, and 7 FTD-*GRN*. * = *p* < 0.05 and ** = *p* < 0.1 by Tukey’s post-hoc test. GCase = β-glucocerebrosidase, Endo = Endoglycosidase H, PNG = PNGase F

We next assessed GCase protein levels by immunoblot with two GCase antibodies, both of which revealed lower levels of mature GCase protein in patients with FTD-*GRN* than controls (Fig. 2a–c). This reduction in GCase differed distinctly from the potentially compensatory increases in HexA and other lysosomal proteins in the same samples. Immunoblots with a rabbit polyclonal anti-GCase antibody revealed an abnormal lower molecular weight band (Fig. 2a) in all but one of the patients with FTD-*GRN* (the exception being case #7 in Table 1, which was unique as further discussed below). We hypothesized that this band might be composed of incompletely glycosylated GCase, indicative of abnormal GCase processing. Under this hypothesis, the opposite directionality of GCase relative to other lysosomal proteins would be due to impairment of GCase processing that prevents a compensatory increase in GCase, such as we have previously observed in *Grn*^*+/−*^ mice [3].

To test this hypothesis, we treated tissue lysates with the deglycosylating enzymes Peptide-*N*-glycosidase F (PNGase F) or Endoglycosidase H (Endo H) and immunoblotted the reaction products with the polyclonal GCase antibody (Fig. 2d). PNGase F removes most N-linked glycans from glycosylated proteins, resulting in a shift to lower molecular weight in SDS-PAGE. With PNGase F treatment, both the normal- and lower molecular weight GCase bands shifted to a single band just above 50 kDa, confirming that the lower molecular weight band in FTD-*GRN* patients was composed of incompletely glycosylated GCase. There were no group differences in the density of the completely deglycosylated GCase band (Fig. 2e), showing that brains from FTD-*GRN* patients contain a similar amount of total GCase protein as controls, but less fully glycosylated, active GCase (Figs. 1f and 2a–c).

In contrast to PNGase F, Endo H is only capable of removing immature glycans that have not undergone final processing in the Golgi apparatus. Thus, increased Endo H-sensitivity is an indicator that a protein may have been misfolded and retained in the endoplasmic reticulum. Neither the normal or lower molecular weight GCase bands were fully Endo H-sensitive in FTD-*GRN* samples (Fig. 2d, defined as shifting to the completely deglycosylated band), though Endo H treatment produced a small shift in the mature GCase band in all samples, including controls. The lower molecular weight GCase band seen in FTD-*GRN* (red arrowhead in Fig. 2d) was roughly the same size as Endo H–treated GCase, consistent with an incomplete glycosylation pattern. Taken together, the PNGase F and Endo H data indicate that impaired GCase activity in FTD-*GRN* patients is due to abnormal GCase processing with incomplete glycosylation.

As a positive control for these experiments, we analyzed GCase immunoblots from fibroblasts carrying the L444P mutation in *GBA* (the gene encoding GCase) which causes the lysosomal storage disorder Gaucher disease in homozygous carriers [27]. The *GBA* L444P mutation produces misfolded GCase that is retained in the endoplasmic reticulum and is incompletely glycosylated [7]. We observed nearly complete loss of mature, glycosylated GCase in homozygous L444P fibroblasts and accumulation of lower molecular weight, incompletely glycosylated GCase in heterozygous and homozygous L444P fibroblasts (Additional file 2: Figure S2), thus confirming that the polyclonal GCase antibody can detect both fully and incompletely processed GCase.

To further investigate abnormal GCase processing, we measured GCase in the soluble and insoluble fractions of brains from controls and FTD-*GRN* patients using a standard sarkosyl-soluble/insoluble fractionation protocol [17] (Fig. 2f–h). We detected normal molecular weight GCase primarily in the sarkosyl-soluble fraction. However, the lower molecular weight, incompletely glycosylated form of GCase was enriched in the sarkosyl-insoluble fraction of 6 of the 7 FTD-*GRN* brains, indicating accumulation of insoluble, immature GCase. In contrast, only one of the control patients accumulated readily detectable amounts of insoluble GCase (Fig. 2g, Additional file 3: Figure S3a).

### A unique *GRN* carrier without a clinical or pathological FTD spectrum diagnosis

Only one of the seven patients with a *GRN* mutation completely lacked the abnormal low-molecular weight GCase band (Additional file 3: Figure S3a). This patient also had the least severe deficits in GCase activity and protein levels, which were within the normal range (Additional file 3: Figure S3b–d). Interestingly, this case (#7 in Table 1) was also clinically and pathologically unique. It was the only *GRN* case without a primary clinical diagnosis of FTD, having features most consistent with dementia with Lewy bodies (DLB). Consistent with that clinical diagnosis, this patient had a primary neuropathological diagnosis of diffuse neocortical Lewy body disease, with only the beginnings of FTLD-TDP type A pathology (Table 1, Additional file 3: Figure S3e). This patient thus represents a rare case of a *GRN* carrier coming to autopsy before FTD symptoms or robust FTLD-TDP pathology manifested, having died from DLB. While this is a single case, it is notable that the only FTD-*GRN* patient in which GCase processing deficits were absent also had the mildest TDP-43 pathology.

### Loss of neuronal GCase in FTD-*GRN*

**Fig. 3:**
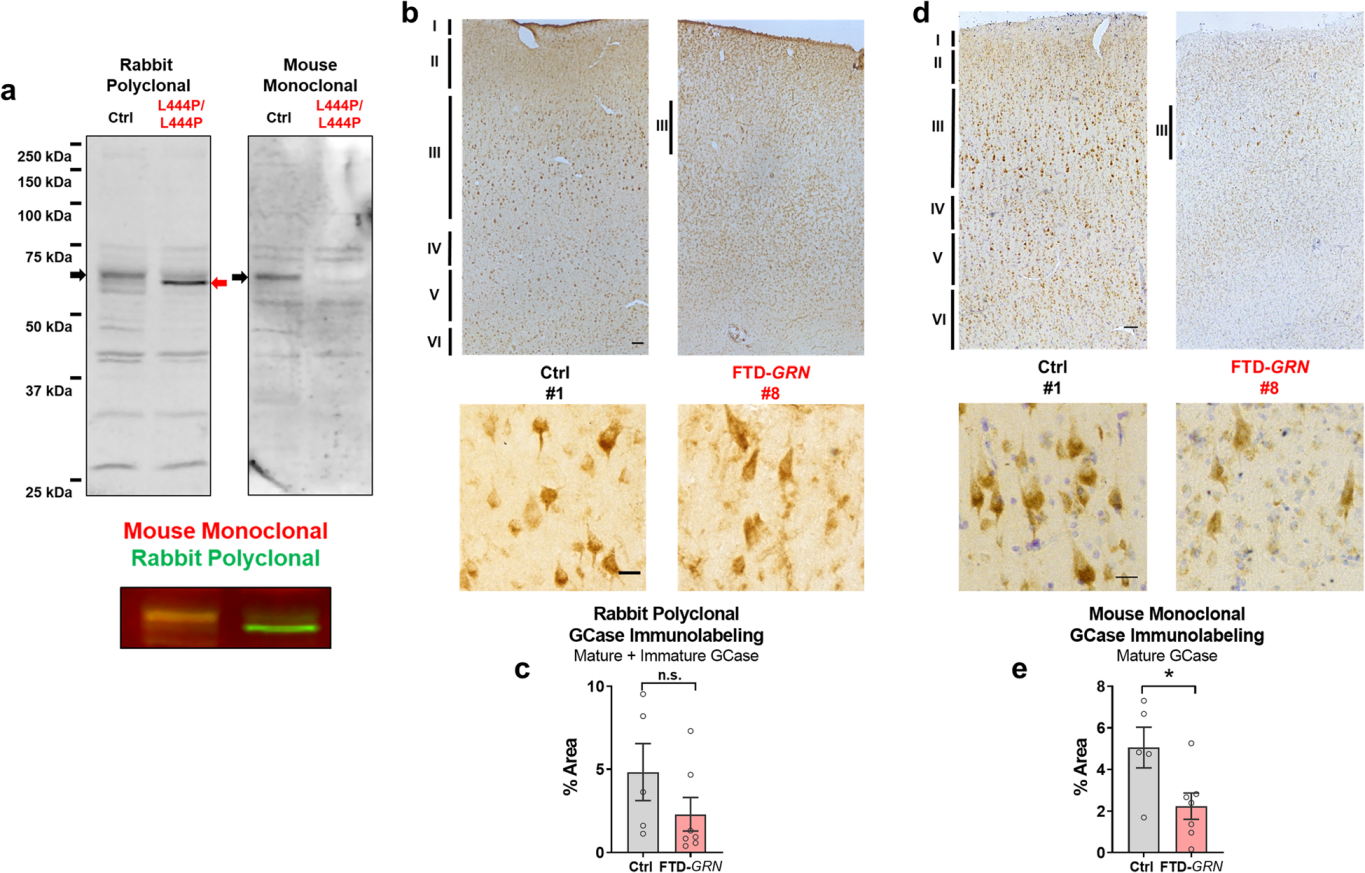
Lower GCase Immunolabeling in FTD-*GRN*. We performed GCase immunostaining with the rabbit polyclonal antibody used in the preceding biochemical experiments and a second mouse monoclonal antibody. **a**, using homozygous *GBA* L444P fibroblasts, we confirmed that the rabbit polyclonal antibody detected an immature form of GCase (also see Additional file 2: Figure S2), but the mouse monoclonal antibody was selective for mature, fully glycosylated GCase. We analyzed GCase immunolabeling in each group in layer III, which contained many strongly-labeled pyramidal neurons in controls. **b**, **c**, FTD-*GRN* patients did not exhibit significantly less GCase immunolabeling with the rabbit polyclonal antibody (*t* test, *p* = 0.2042). **d**, **e**, In contrast, FTD-*GRN* patients had significantly less GCase immunolabeling with the mouse monoclonal antibody selective for mature GCase (*t* test, *p* = 0.0285). Low power images of GCase immunoreactivity throughout the cortex are shown in **b** and **d**, with 100 μm scale bars, with representative 20x images of GCase immunostaining below with a 20 μm scale bars. The numbers for each image reference the patients described in Table 1

We next conducted GCase immunostaining to assess which cell types are affected by the loss of GCase in FTD-*GRN* patients. We immunostained sections of inferior frontal gyrus with the rabbit polyclonal GCase antibody used in the preceding experiments, as well as a second mouse monoclonal GCase antibody. In a preliminary study with L444P Gaucher fibroblasts, we confirmed that the rabbit polyclonal antibody detected both mature, fully glycosylated and immature, incompletely glycosylated GCase, but the mouse monoclonal antibody selectively detected mature, fully glycosylated GCase, not recognizing the lower molecular weight band (Fig. 3a). Immunostaining of control sections revealed similar patterns of immunolabeling with each antibody (Fig. 3b, d), with particularly strong labeling of layer III pyramidal neurons. We therefore measured GCase immunolabeling in 20X images of layer III. Consistent with western blot data (Fig. 2d), FTD-*GRN* patients did not have a significant reduction in total GCase immunolabeling with the rabbit polyclonal antibody (Fig. 3b, c). In contrast, the mouse monoclonal antibody selective for mature GCase revealed a clear reduction in mature.

GCase immunolabeling (Fig. 3d, e). With both antibodies, there appeared to be a loss of strongly GCase-immunolabeled layer III pyramidal neurons, which is consistent with layer III neuronal loss in FTD. We therefore performed additional experiments to determine if the GCase deficits in FTD-*GRN* patients were driven by neuronal loss.

**Fig. 4:**
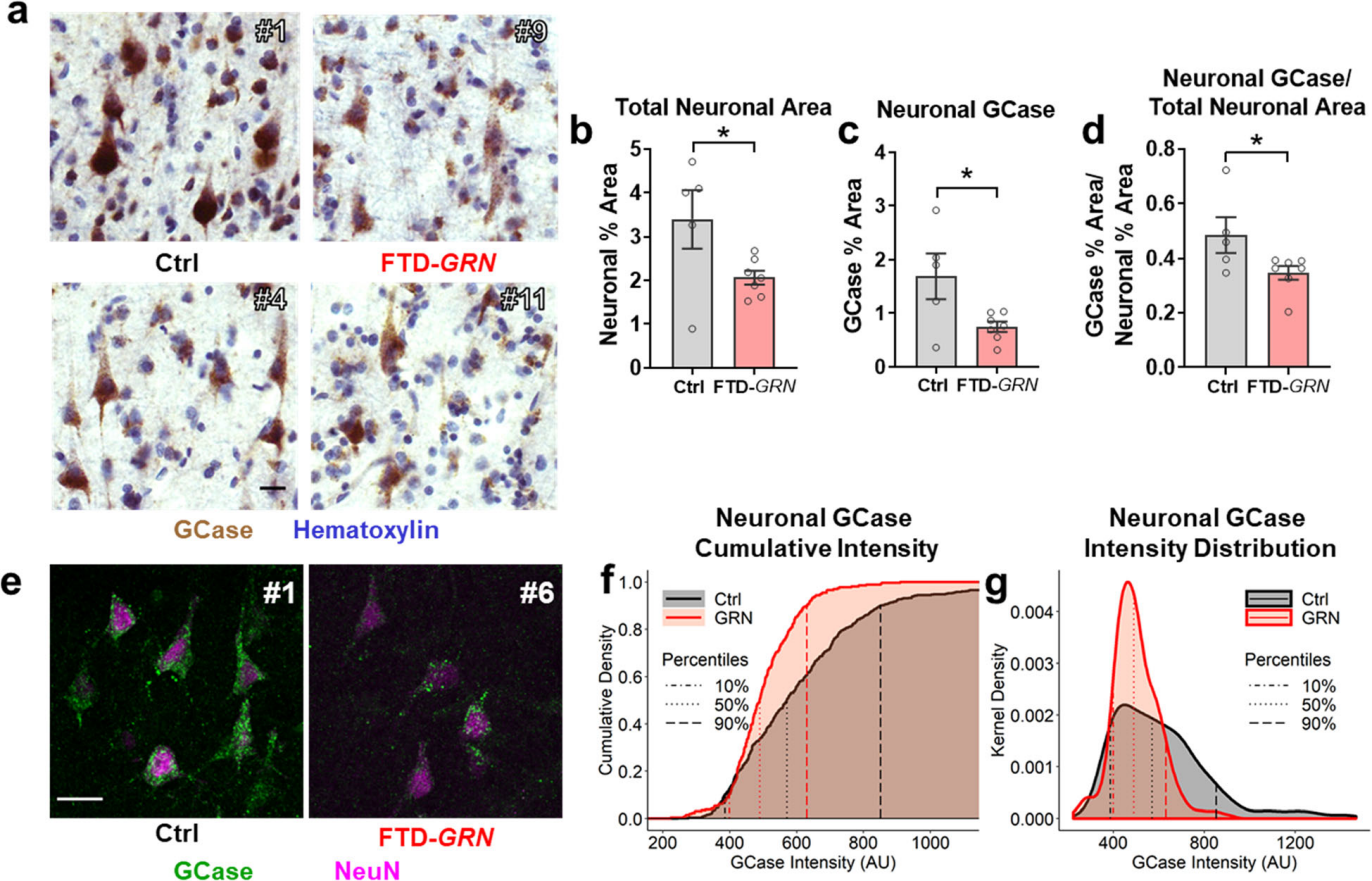
Loss of Neuronal GCase in FTD-*GRN*. **a**, GCase immunostained sections were counterstained with hematoxylin to identify neurons, which revealed both fewer neurons (*t* test, *p* = 0.0437) and less neuronal area (**b**, *t* test, *p* = 0.0458) in layer III of FTD-*GRN* patients than controls, consistent with neurodegeneration. **c**, Within these hematoxylin-labeled neurons, FTD-*GRN* patients had less GCase immunolabeling than controls (*t* test, *p* = 0.031). This observation survived correction for the lower total neuronal area (**d**, *t* test, *p* = 0.048), indicating that the reduced GCase in FTD-*GRN* brains is unlikely to be explained solely by neuronal loss. *n* = 5 controls and 7 FTD-*GRN* patients. **e**, The intensity of fluorescent GCase immunolabeling in cortical neurons was also reduced in FTD-*GRN* patients, with quantification of neuronal GCase intensity shown in **f**, cumulative probability plots and **g**, density plots. These data were analyzed first by Kolmogorov-Smirnov test, showing significantly lower neuronal GCase in FTD-*GRN* (*p* < 0.0001). A more conservative mixed-model regression of the fluorescent intensity distribution data confirmed that the distribution of FTD-*GRN* neurons was skewed toward less intense labeling than controls, with significant group differences found in the 60th (*p* = 0.007), 70th (*p* = 0.001), 80th (*p* = 0.005) and 90th (*p* = 0.022) deciles. *n* = 519 neurons from 5 controls and 262 neurons from 6 FTD-*GRN* patients. All images are labeled with the corresponding case number from Table 1. Representative 40X images of GCase and NeuN immunostaining are shown in **e** with a 20 μm scale bar

To determine if the loss of neuronal GCase in FTD-*GRN* patients was simply a consequence of neuronal loss, we employed two approaches to assess GCase in surviving neurons of FTD-*GRN* patients. First, we used hematoxylin counterstaining to identify pyramidal neurons in sections stained for mature GCase with the mouse monoclonal antibody (Fig. 4a). As expected, FTD-*GRN* patients had fewer hematoxylin-labeled neurons than controls, resulting in a lower total neuronal area (Fig. 4b). ROIs were drawn around hematoxylin-labeled neurons to measure neuronal GCase immunolabeling, revealing that FTD-*GRN* patients had less neuronal GCase immunolabeling than controls (Fig. 4c). This reduced neuronal GCase immunolabeling survived correction for the reduced neuronal area of FTD-*GRN* patients (Fig. 4d). This indicates that while neuronal loss likely contributes to some loss of GCase activity in FTD-*GRN* patients, the surviving neurons still have less GCase immunolabeling than controls.

Second, we assessed neuronal GCase immunolabeling with fluorescent microscopy. Neurons were identified by NeuN immunoreactivity, which labeled both the nucleus (strongly) and soma (faintly) of pyramidal neurons (Fig. 4e) [40]. We measured the mean intensity of GCase immunolabeling in ROIs drawn around NeuN-positive neurons and obtained measurements from 80 to 147 neurons per patient from 5 controls and 11–132 neurons per patient from 6 FTD-*GRN* patients. Initial analyses of these data revealed lower GCase immunolabeling in FTD-*GRN* patients than controls, both by comparing the average intensity of all neurons measured (*t* test, *p* < 0.001) and by comparing the distribution of GCase intensity between neurons from controls and FTD-*GRN* patients (Fig. 4f, g, Kolmogorov-Smirnov test, *p* < 0.0001). We then conducted a follow-up analysis to determine if this difference was driven by true group differences, instead of by the greater number of neurons detected in controls or by variability in the neurons from specific patients. This subsequent analysis was performed with a mixed-model regression to account for serial correlation within subjects, then with undersampling as a sensitivity assessment to limit the influence of any one patient. This mixed-model analysis confirmed significant distribution-based differences in fluorescent intensity between groups, with the control distribution being significantly skewed toward higher intensity relative to FTD-*GRN* patients (Fig. 4f, g). Undersampling with a limit of no more than 40 neurons per patient yielded similar results with no change in statistical significance (Additional file 4: Figure S4). These data show that FTD-*GRN* patients have less neuronal GCase than controls, and that this decrease cannot be explained entirely by neuronal loss, but is also caused by a reduction in the amount of mature GCase per neuron.

### FTD-*GRN* patients do not accumulate GCase substrates

**Fig. 5:**
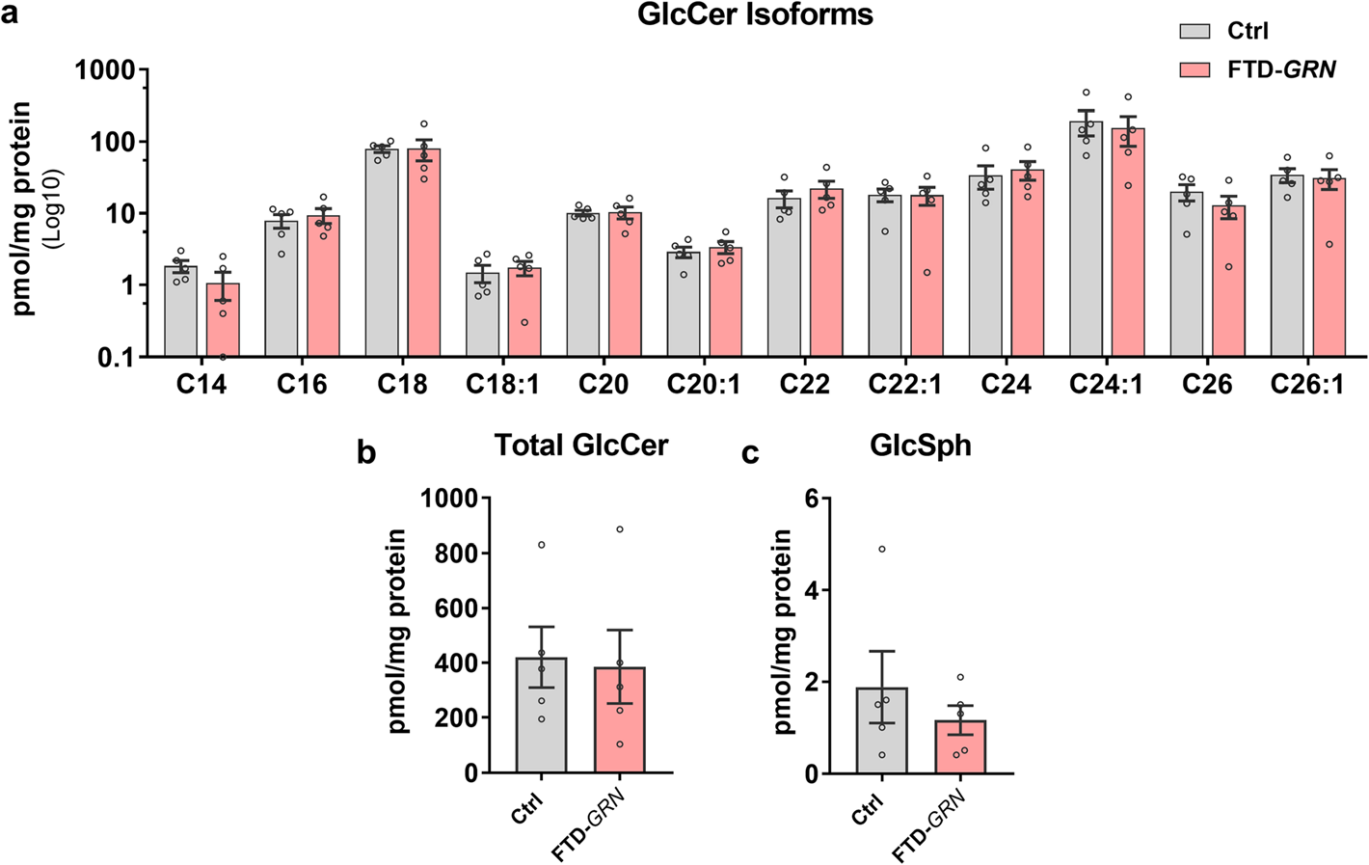
Brains from FTD-*GRN* Patients Do Not Accumulate Glucosylceramide or Glucosylsphingosine. Lipids were extracted from lysates of inferior frontal gyrus of control and FTD-*GRN* patients and analyzed by high performance liquid chromatography/mass spectrometry. No group differences were detected in glucosylceramide isoforms (**a**, MANOVA effect of group, *p* = 0.954), total levels of glucosylceramide (**b**, *t* test, *p* = 0.8470), or glucosylsphingosine (**c**, *t* test, *p* = 0.4199). *n* = 5 controls and 5 FTD-*GRN*. GlcCer = glucosylceramide, GlcSph = glucosylsphingosine

Given the loss of GCase activity in the brain, we tested whether FTD-*GRN* patients accumulated the GCase substrates glucosylceramide (GlcCer) and glucosylsphingosine (GlcSph), which accumulate in the brain of patients with neuronopathic Gaucher disease due to severe GCase deficiency [46, 49]. We did not observe accumulation of any GlcCer isoforms (Fig. 5a), total GlcCer (Fig. 5b), or GlcSph (Fig. 5c) in FTD-*GRN* patients. This is consistent with data from heterozygous *GBA* mutation carriers [9, 22], and indicates that the remaining GCase activity is sufficient to prevent accumulation of these lipids.

### Progranulin and GCase interact

**Fig. 6:**
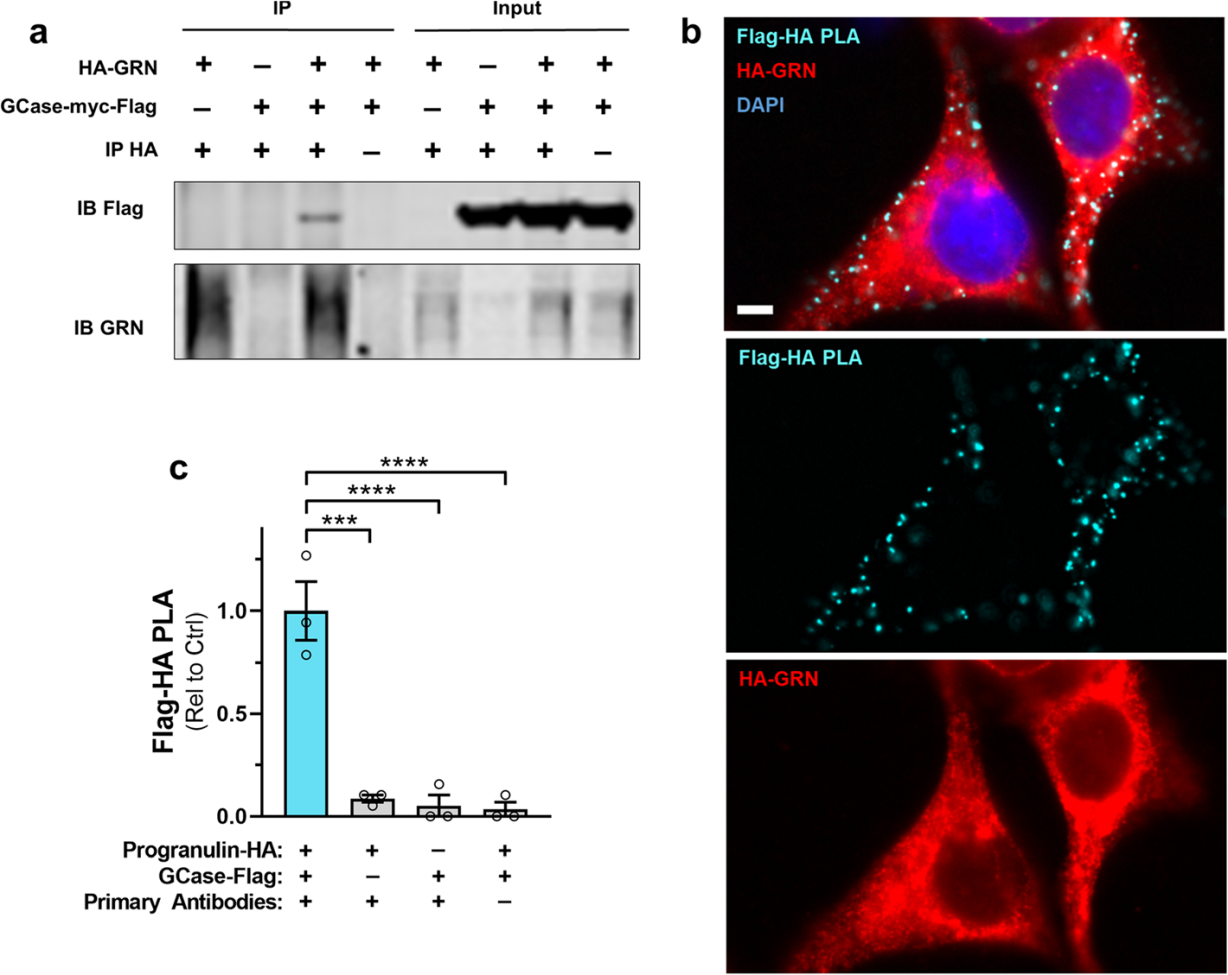
Progranulin Interacts with GCase. HEK-293 cells were co-transfected with constructs expressing HA-tagged human progranulin and/or myc-flag-tagged human GCase. HA-tagged progranulin was then immunoprecipitated from cell lysates with an anti-HA antibody. **a**, Flag-tagged GCase co-immunoprecipitated with progranulin, indicating interaction of the two proteins. **b**, Consistent with the co-immunoprecipitation of GCase with progranulin, we detected strong proximity ligation (PLA) signal in HEK-293 cells co-transfected with human progranulin and human GCase constructs. **c**, The specificity of the Flag-HA PLA signal was confirmed by the presence of significantly more PLA puncta from cells co-transfected with the progranulin-HA and GCase-Flag constructs than in cells transfected with only one of the constructs, or from cells that underwent PLA in the absence of the HA and Flag antibodies (ANOVA effect of experimental condition, *p* < 0.0001, *** = *p* < 0.001 and **** = *p* < 0.0001 by Dunnett’s post-hoc test). The scale bars represents 5 μm. GCase = β-glucocerebrosidase, GRN = progranulin, PLA = proximity ligation assay

Progranulin has recently been reported to interact with GCase and facilitate proper GCase processing and trafficking to the lysosome [29, 30]. Our findings are consistent with these data, so we next sought to confirm that progranulin interacts with GCase. We co-transfected HEK-293 cells with constructs expressing HA-tagged human progranulin (HA-GRN) and/or myc-Flag-tagged human GCase (GBA-myc-Flag), and immunoprecipitated human progranulin from lysates of these cells with an HA-tag antibody. Consistent with a prior report [29], we found that GCase co-immunoprecipitated with progranulin (Fig. 6a).

We next performed in situ analysis of progranulin and GCase interaction by proximity ligation assay (PLA). HEK-293 cells were transfected with HA-GRN and GBA-myc-Flag constructs and processed for PLA 48 h later. We detected abundant PLA signal in cells transfected with both constructs and probed with HA and Flag antibodies (Fig. 6b). We detected essentially no PLA signal in negative control cells transfected with only one of the constructs, or when omitting the primary antibodies on cells transfected with both constructs (Fig. 6c).

### GCase activity deficits in *Grn*^*−/−*^ mice

**Fig. 7:**
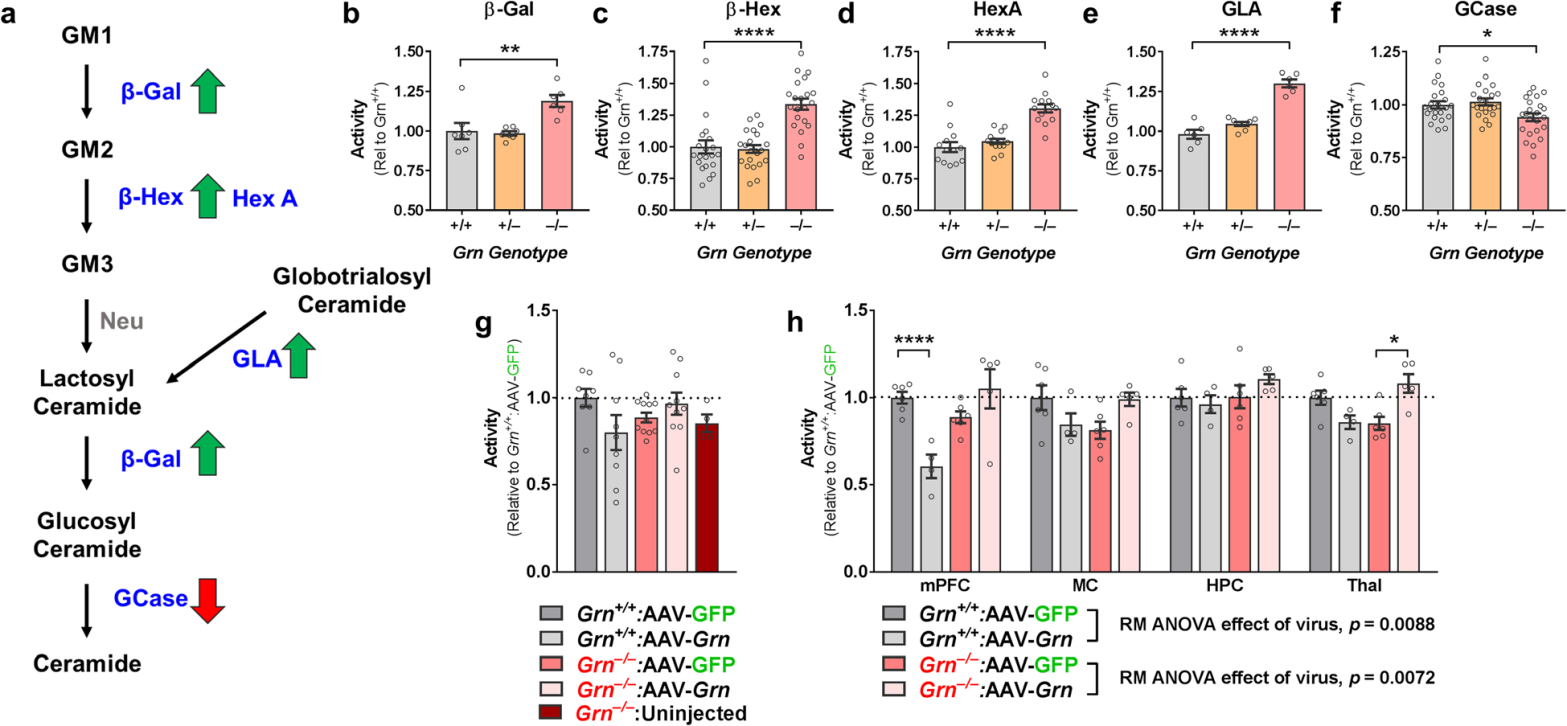
*Grn*^*−/−*^ Mice Have GCase Activity Deficits That Are Corrected by Restoration of Progranulin with an AAV Vector. Frontal cortical lysates from 7 to 10 month-old wild-type, *Grn*^*+/−*^_,_ and *Grn*^*−/−*^ littermates were analyzed for activity of GSL-metabolizing enzymes. A simplified diagram of ganglioside metabolism, with a summary of observed phenotypes in *Grn*^*−/−*^ mice is shown in **a**. Lipids are shown in black, with the enzymes that metabolize each lipid shown in blue. GM1, 2, and 3 = GM1, 2, and 3 ganglioside, β-Gal = β-galactosidase, β-Hex = β-Hexosaminidase, HexA = β-Hexosaminidase A, GLA = α-galactosidase A, GCase = β-glucocerebrosidase, Neu = neuraminidase (activity not measured in this study). **b**–**f**, At this age, *Grn*^*−/−*^ mice exhibited significant increases in activity of β-Gal (**b**, ANOVA, *p* = 0.0024), β-Hex (**c,** ANOVA, *p* < 0.0001), HexA (**d**, ANOVA, *p* < 0.0001), and GLA (**e**, ANOVA, *p* < 0.0001). However, *Grn*^*−/−*^ mice exhibited a decrease in GCase activity (**f**, ANOVA, *p* = 0.0099). *n* = 6–24 per genotype. * = *p* < 0.05 ** = *p* < 0.1, **** = *p* < 0.0001 by Dunnett’s post-hoc test. **g**, Analysis of ventral striatum samples from a cohort of *Grn*^*−/−*^ mice treated with AAV-GFP or AAV-progranulin in the medial prefrontal cortex revealed that restoring progranulin to *Grn*^*−/−*^ mice normalized GCase deficits (ANOVA genotype x treatment interaction, *p* = 0.0122), but that AAV-*Grn* reduced GCase activity in wild-type mice. **h**, Analysis of multiple brain regions from a second group of AAV-treated mice produced similar results, (RM ANOVA, genotype x virus x brain region, *p* = 0.042). Subsequent tests of the effects of virus within genotype confirmed the opposite effects of AAV-*Grn* in each genotype. In wild-type mice, AAV-*Grn* reduced GCase activity (RM ANOVA effect of virus, *p* = 0.0088), but in *Grn*^*−/−*^ mice, AAV-*Grn* increased GCase activity to near wild-type levels (RM ANOVA effect of virus, *p* = 0.0072). * = *p* < 0.05 and **** = *p* < 0.0001 by Sidak’s post-hoc test. *n* = 8–11 mice per group in **g**, with 4 uninjected *Grn*^*−/−*^ mice included as a reference, and 4–6 mice per group in **h**. β-Gal = β-galactosidase, β-Hex = β-Hexosaminidase, HexA = β-Hexosaminidase A, GLA = α-galactosidase A, GCase = β-glucocerebrosidase, Neu = neuraminidase (activity not measured in this study)

To assess the effects of progranulin insufficiency on GCase activity independent of neurodegeneration in an experimentally manipulable model, we measured activity of GCase and other sphingolipid-metabolizing enzymes in frontal cortical lysates from 7 to 10-month-old progranulin-insufficient mice. Except for GCase, *Grn*^*−/−*^ mice had elevated activity of every enzyme measured (β-Gal, β-Hex (total), HexA, and GLA, Fig. 7b–e). Similar to FTD-*GRN* patients, *Grn*^*−/−*^ mice had reduced GCase activity (Fig. 7f). We did not observe any significant changes in enzyme activity in *Grn*^*+/−*^ mice, although we previously observed that older *Grn*^*+/−*^ mice (12–20 months) exhibit elevated HexA and GCase activity [3]. These data indicate that progranulin insufficiency impairs cortical GCase activity in the absence of neuronal loss and indicate that GCase requires progranulin for normal activity in the brain.

### Progranulin gene therapy normalizes GCase activity in *Grn*^*−/−*^ mice

Since progranulin insufficiency impairs GCase activity in the brain, we hypothesized that restoration of progranulin would normalize GCase activity. We have previously shown that AAV-mediated restoration of progranulin to *Grn*^*−/−*^ mice reduces lipofuscinosis and normalizes elevated cathepsin D activity [4]. We therefore analyzed GCase activity in tissue samples from the mice in our previous study [4] to determine if restoration of progranulin could normalize GCase deficits. These mice were treated with AAV-progranulin (AAV-*Grn*) or AAV-GFP by infusion into the medial prefrontal cortex at 10–12 months of age and euthanized 8–10 weeks later for brain collection. We began our investigation of GCase activity with ventral striatum lysates in which we previously observed normalization of cathepsin D activity [4]. We observed a genotype-specific effect of AAV-*Grn*. AAV-*Grn* boosted GCase activity in *Grn*^*−/−*^ mice (Fig. 7g), showing that restoration of progranulin can improve GCase deficits. In contrast, AAV-*Grn* suppressed GCase activity in wild-type mice. This was somewhat surprising but consistent with our prior observations of reduced cathepsin D activity in AAV-*Grn*-treated wild-type mice [4].

To confirm this finding, we analyzed tissue samples from a second group of mice treated with AAV-*Grn* by infusion into the medial prefrontal cortex at 3–6 months of age and euthanized for analysis 4 weeks later (average age at euthanasia = 5.9 months). Similar to the first cohort, we found that restoration of progranulin with AAV-*Grn* gene therapy in *Grn*^*−/−*^ mice normalized GCase deficits across multiple brain regions (Fig. 7h).

## Discussion

This study shows that progranulin insufficiency impairs GCase activity in the frontal cortex of patients with FTD-*GRN*. GCase was the only sphingolipid-metabolizing enzyme among those analyzed in this study to show impaired activity in FTD-*GRN* patients, suggesting a specific GCase deficit, rather than a general deficit in sphingolipid metabolism. We found evidence in FTD-*GRN* patients for abnormal GCase processing, with incomplete glycosylation and accumulation in the sarkosyl-insoluble fraction. GCase impairment in FTD-*GRN* patients is likely the direct result of progranulin insufficiency, as *Grn*^*−/−*^ mice also exhibited impaired GCase activity that was normalized by restoring progranulin with AAV-*Grn* gene therapy.

Brains from FTD-*GRN* patients accumulate lysosomal proteins, possibly due to underlying lysosomal dysfunction [25], and this study indicates that GCase deficits may be a component of the underlying lysosomal dysfunction. These GCase deficits may be caused by impaired GCase processing due to progranulin insufficiency. This work adds to prior reports that progranulin interacts with GCase [29, 30], and recent reports of reduced GCase activity in brains from *Grn*^*−/−*^ mice [72] and iPSC-derived neurons from *GRN* mutation carriers [65], further supporting the requirement of progranulin for normal GCase activity in the brain.

Regulation of lysosomal enzyme activity may be a key function of progranulin. Progranulin also regulates the activity of cathepsin D [6, 11, 12, 64, 71] and HexA [14]. For both cathepsin D and HexA, we and others have observed potentially compensatory increases in expression in FTD-*GRN* patients [25] and progranulin-insufficient mice [3, 24, 25, 33, 61, 62] that stands in contrast to the GCase deficits reported in the present study. Interestingly, we also observed increased GCase activity in a prior study of *Grn*^*+/−*^ mice [3]. This contrast may be explained by a GCase processing deficit that emerges when progranulin levels fall below a certain threshold. Progranulin-insufficient cells may compensate for impaired HexA and CatD activity by increasing HexA and CatD expression, but may be unable to compensate for impaired GCase activity due to impaired GCase processing.

The role of impaired GCase activity in FTD-*GRN* pathogenesis will be an important topic for future investigation. It is notable that the clinical spectrum of patients with *GRN* mutations is quite different from that of patients with *GBA* mutations. Homozygous *GRN* mutation carriers develop the lysosomal storage disorder NCL [2, 31, 59], and heterozygous carriers typically develop dominantly inherited FTD [5, 16], though some develop other disorders such as Alzheimer’s disease, Parkinson’s disease (PD), or DLB [10, 32, 56, 68]. In contrast, homozygous *GBA* mutation carriers develop the lysosomal storage disorder Gaucher disease [26], and heterozygous carriers are at increased risk for PD and DLB [1, 42, 48]. PD and DLB patients without *GBA* mutations also exhibit GCase activity deficits in certain brain regions [15, 21, 39, 55], perhaps due to impairment of GCase activity by α-synuclein [38]. Available data indicate that *GBA* mutations do not increase risk for AD [63]. There is even less data on the influence of *GBA* mutations on FTD. One study reported increased incidence of *GBA* mutations in patients with corticobasal syndrome, but not other FTD subtypes [51]. Based on available data on progranulin’s role in the lysosome, it seems possible that GCase deficits are one of several lysosomal deficits in *GRN* mutation carriers that contribute to disease. Defining progranulin’s lysosomal interactome and the lysosomal deficits induced by progranulin insufficiency may help clarify the role of GCase deficits in FTD-*GRN* pathogenesis.

*GBA* mutations are a major risk factor for PD and DLB [1, 42, 48], so the impairment of GCase activity in FTD-*GRN* patients and *Grn*^*−/−*^ mice raises the question of whether *GRN* mutations or variants may also increase PD and DLB risk. *GRN* mutation carriers occasionally develop PD or DLB [10, 32, 56, 68], though they usually develop an FTD syndrome with TDP-43 pathology. Genetic studies testing for association of *GRN* variants with PD have produced mixed results [13, 28, 34, 47], though a preprint report of a large genome-wide association study has identified *GRN* as a risk gene for PD [41]. Interestingly, PD patients have lower circulating progranulin levels than controls [34]. Gaucher disease patients also have lower levels of serum progranulin than controls and have higher frequencies of *GRN* gene variants associated with lower progranulin levels [30]. In mouse models, progranulin is protective against dopaminergic neuronal loss due to MPTP [37, 66]. This protection has been associated with progranulin’s anti-inflammatory effects. However, it is possible that progranulin’s interaction with GCase could play a role, as both *Grn*^*−/−*^ mice and *Gba*^*+/L444P*^ mice are more sensitive than wild-type mice to MPTP-induced dopaminergic neuronal loss [37, 70]. Thus, while current data on a role for progranulin in PD or DLB are sparse, this may be an interesting topic for future study.

The GCase deficits in *Grn*^*−/−*^ mice show that progranulin insufficiency alone modestly impairs GCase activity in the brain, but we also obtained evidence that disease state may influence GCase activity in *GRN* mutation carriers. It is quite rare for a *GRN* mutation carrier to come to autopsy before developing FTD, as we observed in one case (#7 in Table 1). In this case, which had only the beginnings of FTLD-TDP type A pathology (Additional file 3: Figure S3f), we did not observe the GCase abnormalities that were present in *GRN* cases with FTD-spectrum disorders and FTLD-TDP type A pathology: GCase activity (Additional file 3: Figure S3c) and GCase protein levels (Additional file 3: Figure S3d,e) were within the range of controls, and the low molecular weight GCase band was not observed in either soluble or insoluble fractions (Additional file 3: Figure S3a, b). Interestingly, this patient had a synucleinopathy, with a DLB clinical syndrome and Lewy body disease pathology (Table 1). Synucleinopathies including PD and DLB have been associated with GCase abnormalities, particularly in the substantia nigra [15, 21, 39, 55], so one might have expected to see GCase abnormalities even in the absence of significant FTD or FTLD pathology. However, patients with PD and DLB do not typically exhibit GCase deficits in the frontal cortex [15, 21, 39, 55]. Thus, the lack of GCase deficits in this case are consistent with data on patients with DLB. Although it is a single case, if confirmed in future studies, these observations would suggest that the GCase abnormalities we observed correlate with onset of dysfunction.

## Conclusions

In summary, these data expand our knowledge of the lysosomal dysfunction induced by progranulin insufficiency in the brain. We have shown that progranulin insufficiency impairs GCase activity in the brain, likely by disrupting GCase processing, and that restoration of progranulin corrects these activity deficits in *Grn*^*−/−*^ mice. Future studies will investigate the role of these GCase deficits in FTD pathogenesis.

## Supplementary information

**Additional file 1: Figure S1.** Elevated HexA, LAMP-1, and LAMP-2 in Inferior Frontal Gyrus from FTD-*GRN* Patients

**Additional file 2: Figure S2.** Representative GCase Blots from Gaucher Disease Fibroblasts

**Additional file 3: Figure S3.** Absence of Low-molecular Weight GCase and Lack of GCase Deficits in a *GRN* Carrier with Lewy Body Disease

**Additional file 4: Figure S4.** Undersampled Intensity Data

## Data Availability

All data generated or analyzed during this study are included in this published article.

## Abbreviations

AAV: adeno-associated virus
AD: Alzheimer’s disease
bvFTD: behavioral variant frontotemporal dementia
CBS: corticobasal syndrome
DLB: dementia with Lewy bodies
FTD: frontotemporal dementia
GCase: β-glucocerebrosidase
GLA: α-galactosidase A
Grn: progranulin
GSL: glycosphingolipid
HexA: β-Hexosaminidase A
NCL: neuronal ceroid lipofuscinosis
Neu: neuraminidase
nfPPA: nonfluent variant primary progressive aphasia
PD: Parkinson disease
PLA: proximity ligation assay
PMI: postmortem interval
β-Gal: β-galactosidase
β-Hex: β-Hexosaminidase
