# Supplementary material for: Impaired β-glucocerebrosidase activity and processing in frontotemporal dementia due to progranulin mutations
Source: Acta Neuropathol Commun. 2019 Dec 23;7:218. doi: 10.1186/s40478-019-0872-6 (PMC6929503; doi:10.1186/s40478-019-0872-6)
Supplement: Supplementary file 1 — Additional file 1: Figure S1. Elevated HexA, LAMP-1, and LAMP-2 in Inferior Frontal Gyrus from FTD-GRN Patients [file 40478_2019_872_MOESM1_ESM.docx]

**
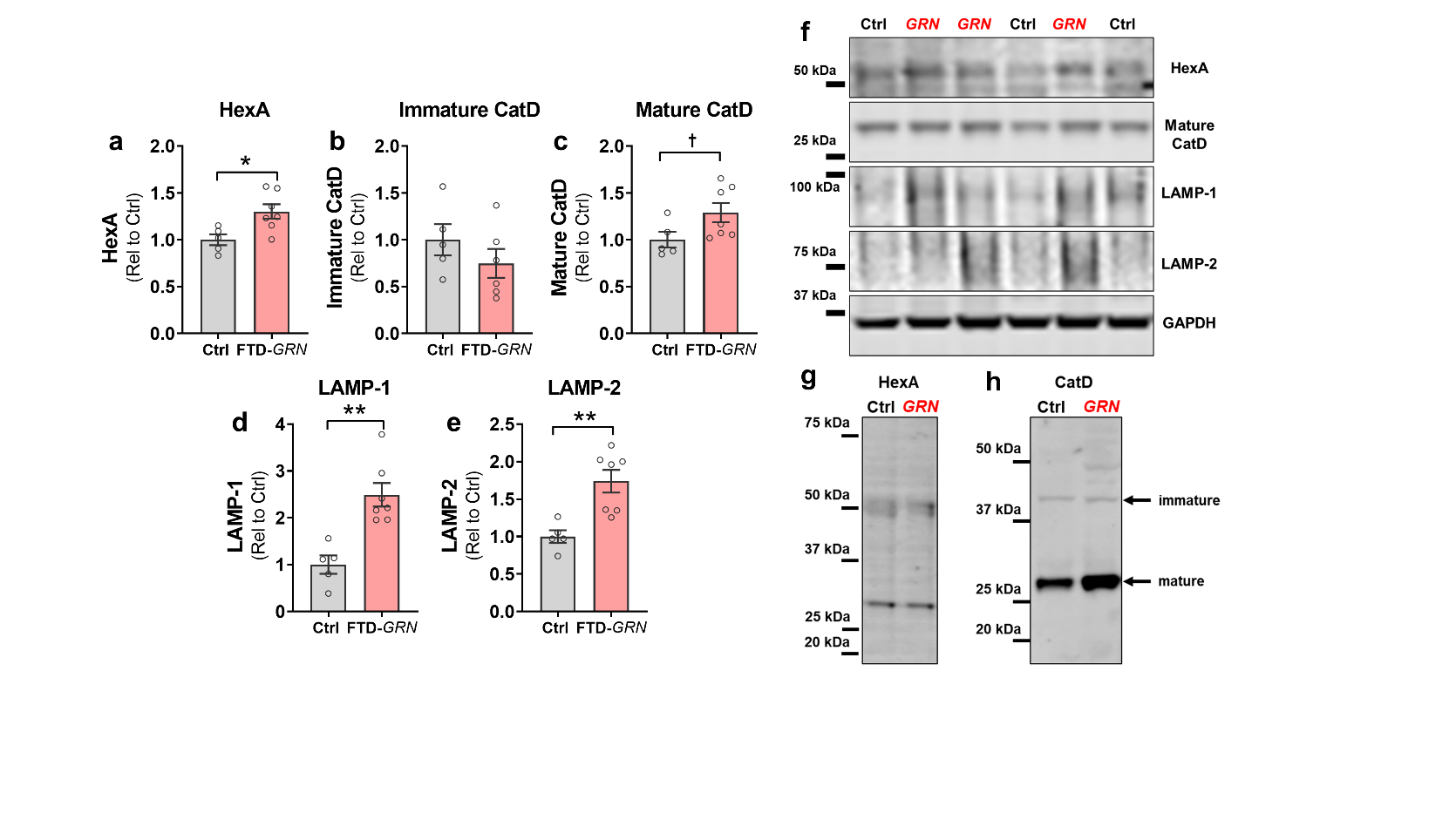
**

**Figure S1 – Elevated HexA, LAMP-1, and LAMP-2 in Inferior Frontal Gyrus from FTD-*GRN* Patients.**

**a**, The elevated HexA activity of FTD-*GRN* patients was associated with higher levels of HexA protein (*t* test, *p* = 0.0172). **b**, While immature cathepsin D (CatD) did not differ between groups (*t* test, *p* = 0.2948), FTD-*GRN* patients also had a trend for elevated levels of mature CatD (**c**, *t* test, *p* = 0.0665), and **d**,**e**, significantly elevated levels of LAMP-1 (*t* test, *p* = 0.0014) and LAMP-2 (*t* test, *p* = 0.0035) relative to controls. Representative blots are shown in **f**, with longer images of HexA and CatD blots shown in **g** and **h**. n = 5 controls and 6-7 FTD-*GRN*.
