# Supplementary material for: Impaired β-glucocerebrosidase activity and processing in frontotemporal dementia due to progranulin mutations
Source: Acta Neuropathol Commun. 2019 Dec 23;7:218. doi: 10.1186/s40478-019-0872-6 (PMC6929503; doi:10.1186/s40478-019-0872-6)
Supplement: Supplementary file 2 — Additional file 2: Figure S2. Representative GCase Blots from Gaucher Disease Fibroblasts [file 40478_2019_872_MOESM2_ESM.docx]

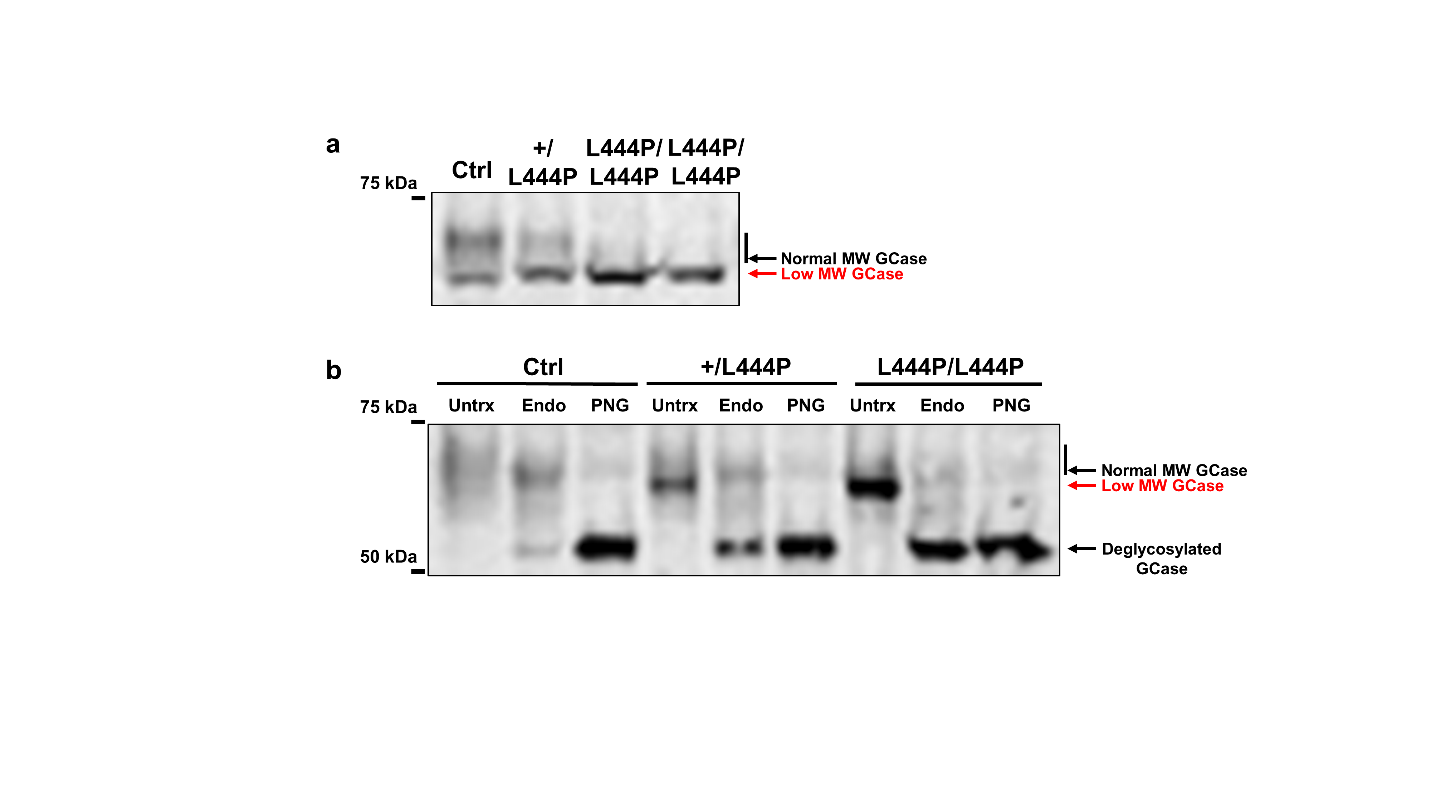


**Figure S2** **– Representative GCase Blots from Gaucher Disease Fibroblasts.**

GCase in a control fibroblast line (Coriell #GM00730), a heterozygous *GBA* L444P line (#GM00878), and two homozygous *GBA* L444P lines (#GM08760 and GM00877), was analyzed by western blot using the rabbit polyclonal GCase antibody. **a**, In all lines, GCase was more heavily glycosylated and ran at a higher apparent molecular weight than in brain samples. As observed in FTD-*GRN* cases, lines carrying the L444P mutation had a more prominent low-molecular weight GCase band than the control line. **b**, Unlike brain samples, this low-molecular weight band was sensitive to Endo H, indicating that it was comprised of immature GCase that did not exit the endoplasmic reticulum. Endo = endoglycosidase H, PNG = PNGase F.
