# Supplementary material for: Impaired β-glucocerebrosidase activity and processing in frontotemporal dementia due to progranulin mutations
Source: Acta Neuropathol Commun. 2019 Dec 23;7:218. doi: 10.1186/s40478-019-0872-6 (PMC6929503; doi:10.1186/s40478-019-0872-6)
Supplement: Supplementary file 3 — Additional file 3: Figure S3. Absence of Low-molecular Weight GCase and Lack of GCase Deficits in a GRN Carrier with Lewy Body Disease [file 40478_2019_872_MOESM3_ESM.docx]

**
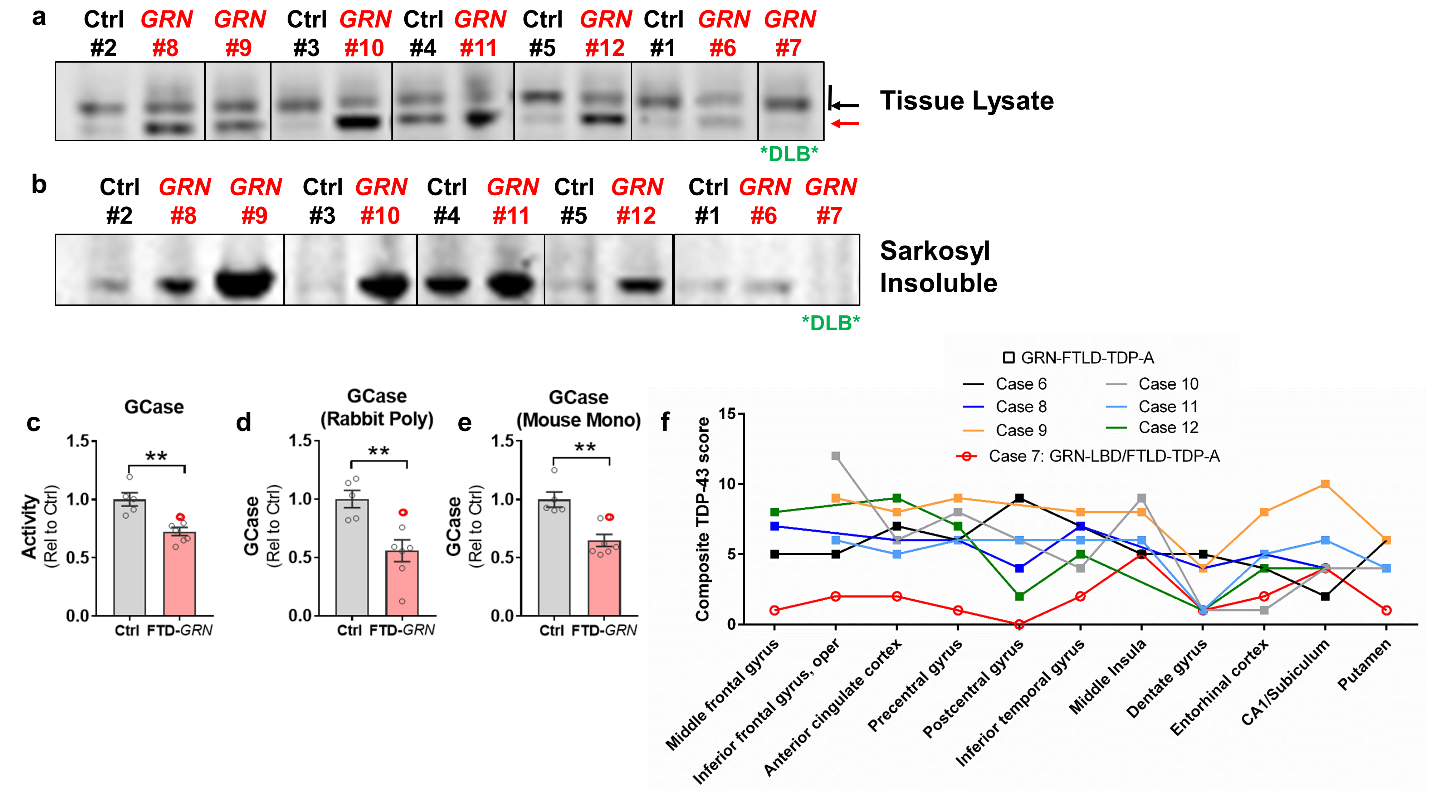
Figure S3** **– Absence of Low-molecular Weight GCase and Lack of GCase Deficits in a *GRN* Carrier with Lewy Body Disease.**

A *GRN* patient with an atypical syndrome resembling DLB (case 7, Table 1) and diffuse neocortical Lewy body disease pathology (marked LBD) was the only *GRN* patient that failed to accumulate low-molecular weight GCase, as shown in representative blots of from brain lysates (**a**) or in blots of sarkosyl insoluble GCase (**b**). **c**–**e**, This patient was in the normal range of both GCase activity and protein levels. (Case 7 is highlighted in red in **c,** data from Fig. 1f, **d,** data from Fig. 2b, and **e,** data from Fig. 2c). **f**, This patient had the mildest FTLD-TDP pathology of the cases studied. In **a**, normal-molecular weight GCase is indicated by the black arrow and bar, and low-molecular weight GCase is indicated by the red arrow. Ctrl = control, *GRN* = FTD-*GRN*, LBD = Lewy body disease, FTLD = frontotemporal lobar degeneration.
