# Supplementary material for: Impaired β-glucocerebrosidase activity and processing in frontotemporal dementia due to progranulin mutations
Source: Acta Neuropathol Commun. 2019 Dec 23;7:218. doi: 10.1186/s40478-019-0872-6 (PMC6929503; doi:10.1186/s40478-019-0872-6)
Supplement: Supplementary file 4 — Additional file 4: Figure S4. Undersampled Intensity Data [file 40478_2019_872_MOESM4_ESM.docx]

**
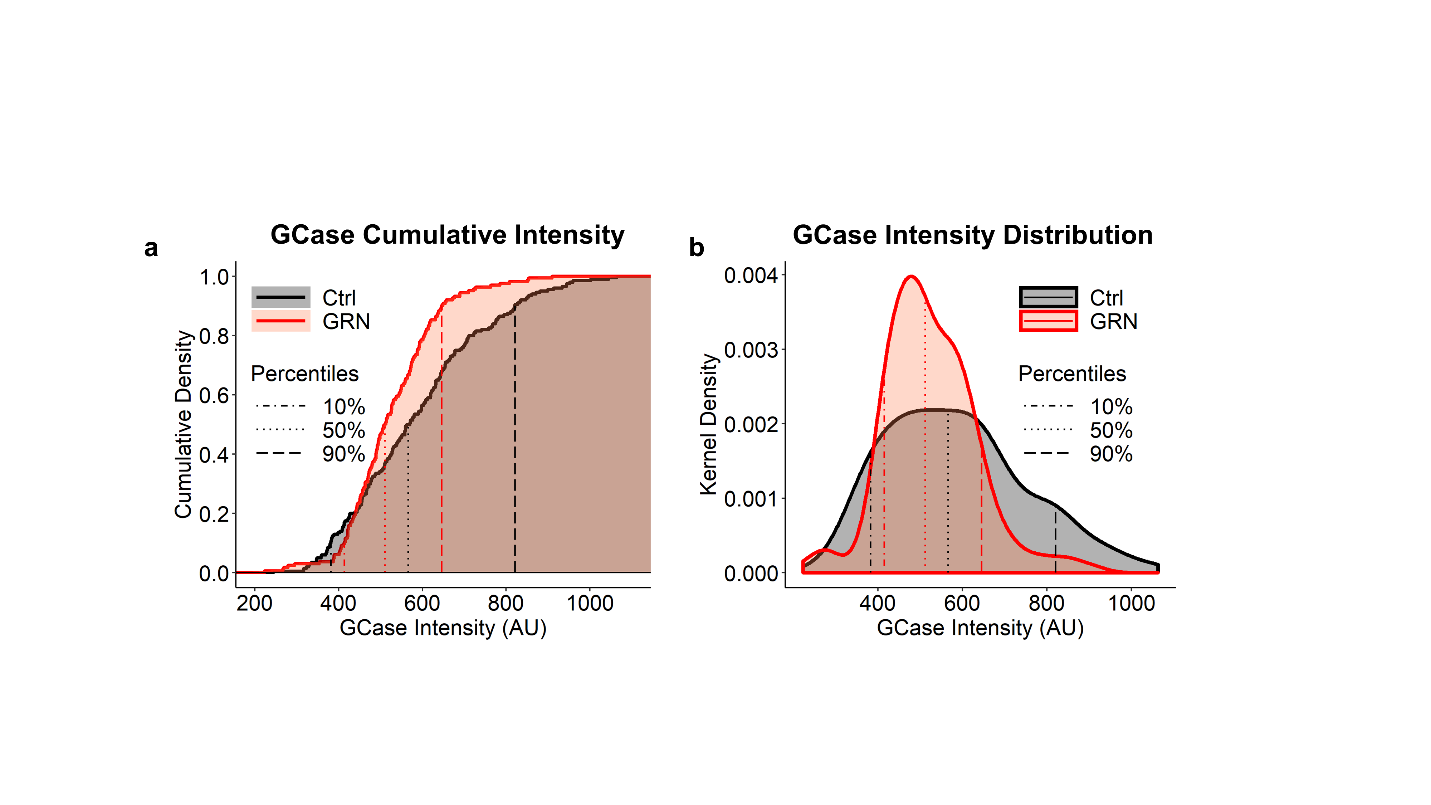
**

**Figure S4** **–Undersampled Intensity Data.**

The undersampled data used for secondary analysis of GCase fluorescent intensity are shown in **a** and **b**. For this dataset, n was capped at a maximum of 40 neurons per patient. All five controls and two FTD-*GRN* patients had more than 40 neurons, but four FTD-*GRN* patients had less than 40 neurons detected (range of 11-27 neurons). For these 4 patients, all available neurons were included in the analysis.
